# Multipulse transcranial electrical stimulation (TES): normative data for motor evoked potentials in healthy horses

**DOI:** 10.1186/s12917-018-1447-7

**Published:** 2018-04-03

**Authors:** Sanne Lotte Journée, Henricus Louis Journée, Cornelis Marinus de Bruijn, Cathérine John Ghislaine Delesalle

**Affiliations:** 1 Equine Diagnostics, Tergracht 2A, 9091 BG Wijns, The Netherlands; 2 Department of Neurosurgery, University Groningen, University Medical Center Groningen, Hanzeplein 1, 9713 GZ Groningen, The Netherlands; 3 Wolvega Equine Clinic, Stellingenweg 10, 8474 EA Oldeholtpade, The Netherlands; 4 Department of Comparative Physiology and Biometrics, Faculty of Veterinary Medicine, Ghent University, Salisburylaan 133, 9820 Merelbeke, Belgium

**Keywords:** Transcranial electric stimulation, TES, TMS, MEP, Neurology, Horses, Spinal cord function

## Abstract

**Background:**

There are indications that transcranial electrical stimulation (TES) assesses the motor function of the spinal cord in horses in a more sensitive and reproducible fashion than transcranial magnetic stimulation (TMS). However, no normative data of TES evoked motor potentials (MEP) is available.

**Results:**

In this prospective study normative data of TES induced MEP wave characteristics (motor latency times (MLT); amplitude and waveform) was obtained from the extensor carpi radialis (ECR) and tibial cranialis (TC) muscles in a group of healthy horses to create a reference frame for functional diagnostic purposes. For the 12 horses involved in the study 95% confidence intervals for MLTs were 16.1–22.6 ms and 31.9–41.1 ms for ECR and TC muscles respectively. Intra-individual coefficients of variation (CV) and mean of MLTs were: ECR: 2.2–8,2% and 4.5% and TC: 1.4–6.3% and 3.5% respectively. Inter-individual CVs for MLTs were higher, though below 10% on all occasions.

The mean ± sd of MEP amplitudes was respectively 3.61 ± 2.55 mV (ECR muscle left) and 4.53 ± 3.1 mV (right) and 2.66 ± 2.22 mV (TC muscle left) and 2.55 ± 1.85 mV (right). MLTs showed no significant left versus right differences.

All MLTs showed significant (*p* < 0.05) voltage dependent decreases with slope coefficients of linear regression for ECR: − 0.049; − 0.061 ms/V and TC: − 0.082; − 0.089 ms/V (left; right). There was a positive correlation found between height at withers and MLTs in all 4 muscle groups. Finally, reliable assessment of MEP characteristics was for all muscle groups restricted to a transcranial time window of approximately 15–19 ms.

**Conclusions:**

TES is a novel and sensitive technique to assess spinal motor function in horses. It is easy applicable and highly reproducible. This study provides normative data in healthy horses on TES induced MEPs in the extensor carpi radialis and tibialis cranialis muscles bilaterally. No significant differences between MLTs of the left and right side could be demonstrated. A significant effect of stimulation voltage on MLTs was found. No significant effect of height at the withers could be found based upon the results of the current study. A study in which both TMS and TES are applied on the same group of horses is needed.

## Background

After transcranial magnetic stimulation (TMS) was introduced in equine medicine by Mayhew and Washborn in 1996, the method evolved into a diagnostic tool used to assess the motor function of the spinal cord in horses [1–3]. Lesions of the spinal cord are mostly characterized by a significant increase of motor latency times (MLT) and a decrease of muscular evoked potential (MEP) amplitudes in response to TMS [4]. The TMS technique is based on the induction of electrical currents by a strong magnetic pulse, created by a coil, which is placed on the forehead of the horse with subsequent activation of axons in the motor cortex taking place [5, 6]. The generated action potentials are relayed by upper motor neurons (UMNs) before further conduction takes place along the motor pathway of the corticospinal tract, the lower motor neurons (LMN) and finally the motor nerves to the skeletal muscles.

Recently, our group has published an alternative stimulation technique: transcranial electrical stimulation (TES) [7].

In contrast to TMS, the TES technique entails direct application of an electrical current to a pair of needle electrodes, which are subcutaneously inserted on the forehead of the horse. In contrast to TMS, the TES technique bypasses predominantly the route via the motor cortex of the brain [8] to the pyramidal tract. This explains why the technique is preferred over TMS to be applied in human patients under generalized anesthesia [9]. Indeed, unlike TMS, TES predominantly targets direct stimulation of the corticospinal tract. Corticospinal axons in humans and primates are oriented perpendicular to the cortical surface. Anodal transcranial stimulation depolarizes corticospinal axons directly and has a lower stimulation threshold than cathodal stimulation [5, 10–12]. These direct action potentials can be recorded as d-waves [7]. Cell bodies of UMNs are predominantly bypassed and less influenced by modulating inputs from the cortex of the brain [8, 13–17]. This is in contrast to the elicited action potentials in the cortex from TMS that are relayed via UMNs and are recorded as indirect waves, i-waves, in the corticospinal tract [18]. In TMS, d-waves are incidentally also generated, however these are less dominant than i-waves [16, 17]. Like in TMS, TES has to be performed in sedated horses [19, 20] while sedation was reported not to affect MLTs [20].

In our previous prospective study, we showed that there are clear indications that the TES technique is less sensitive to cortical function due to direct stimulation of the corticospinal tract [7]. The applied TES multipulse train stimulation protocol helps to overcome hyperpolarization induced by sedation, which is a disadvantage of a single pulse stimulation protocol applied in the TMS technique. Indeed, the multipulse train stimulation protocol is more robust, since each stimulation pulse is able to produce multiple descending volleys of d- and i-waves in the pyramidal tract [11, 12, 21]. These initiate spatial and temporal summations of excitatory postsynaptic potentials (epsp). This summation process from multipulse stimulation results in a powerful depolarization of LMNs.

Normative data on TMS in horses has been published by Nollet et al. [3]. However, no normative data is available for TES in horses. The aim of the current study was 1) to obtain normative data of MLTs, waveform, and amplitudes of TES induced muscular evoked potentials (MEP), as well as earlier reported boundaries of the transcranial time windows [7] in muscle groups of four extremities in a group of healthy horses to create an initial frame of reference for clinical diagnostic purposes and 2) to study the effect of body side (left versus right), stimulation voltage intensity and height at withers of the horses on MEP characterizing parameters.

## Methods

Twelve healthy client owned warmblood horses, consisting of 6 gelding and 6 mares, aged: 10.7 ± 5.5 years (mean ± sd) were included in the study. No abnormalities were found during clinical neurological examination. The height at withers was 160.8 ± 10 cm (mean ± sd). The animal ethics committee of the University of Groningen, The Netherlands approved the study protocol (DEC6440A).

**Fig. 1:**
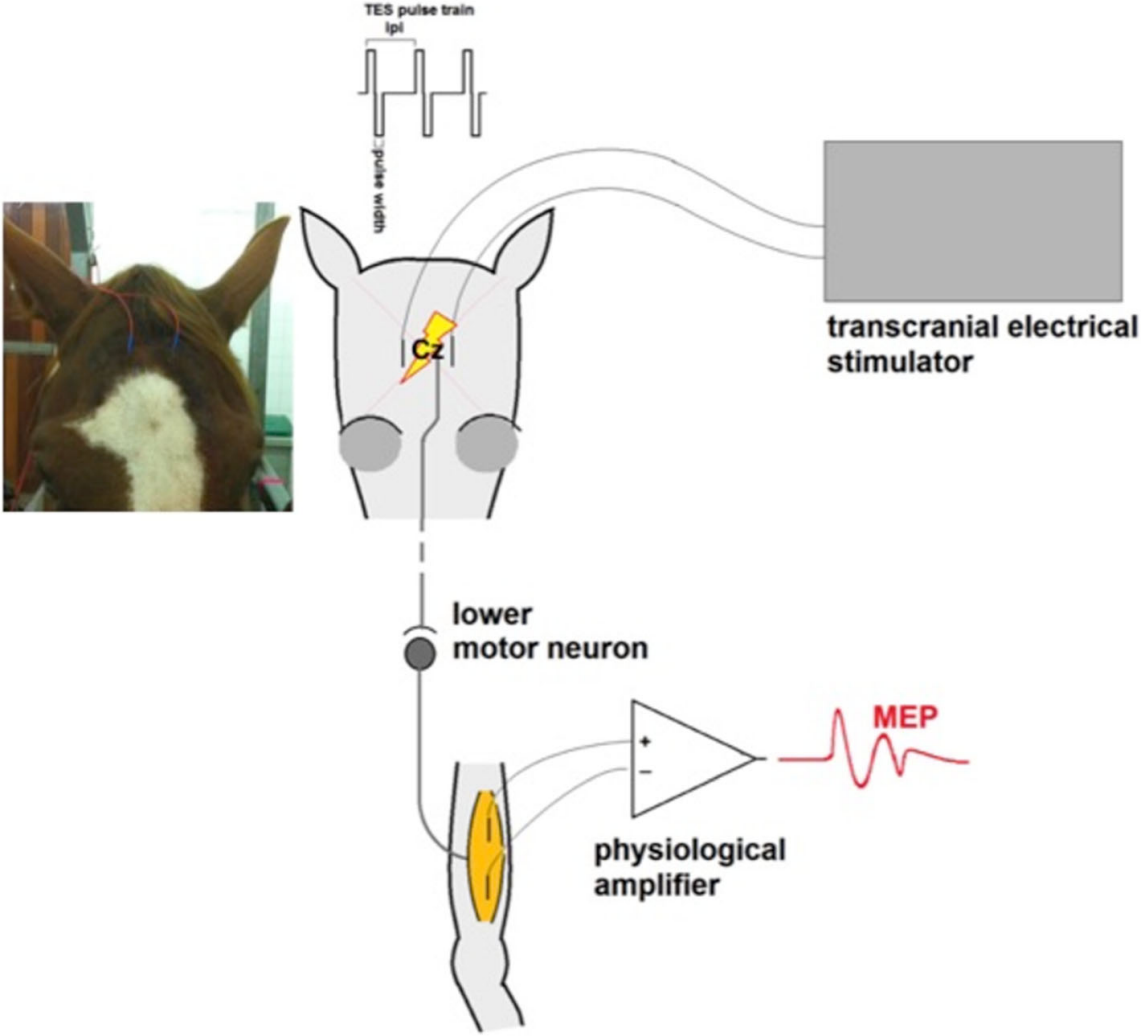
Schematic drawing of the TES-MEP set-up. A transcranial electrical stimulator is connected to subcutaneously inserted needle electrodes bilaterally from the vertex of the skull. A multipulse stimulation consists of 3 biphasic pulses/train, pulse width 0.1 ms/phase, ipi = 1.3 ms. Elicited action potentials in corticospinal axons cross the midline at the decussatio pyramis and are conveyed to LMNs that relay to peripheral motor axons. After passing the neuromuscular junction muscular motor potentials are generated in muscle fibers and recorded at a pair of needle electrodes that are connected to a physiological amplifier

Horses were prepared as previously described [7]. Sedation was performed in all horses (*n* = 12), each time by I.V. administration of detomidine (Detosedan, AST Farma B.V., Oudewater, The Netherlands) and butorphanol (Butomidor, AST Farma B.V., Oudewater, The Netherlands) (both 1.5–2.0 μg/kg bwt in total). Two needle electrodes (L 35 mm, Ø 0.45 mm, type RMN35/0.45 Electrocap BV, Nieuwkoop, Netherlands) were inserted subcutaneously parallel to each other and caudo-rostrally on the forehead (Fig. 1). The needle electrodes were separated 5 cm from each other, with their middle points 2.5 cm bilateral from the central location Cz on the forehead. The horses returned to their owners after completion of the procedure and a final clinical examination.

TES was performed using biphasic multipulse trains using a constant voltage of a human intraoperative neurophysiological monitoring system (Neuro-Guard JS Center, Bedum, The Netherlands). A bandpass filter was used with a high pass filter of 50 Hz, and a low pass filter of 2500 Hz (3 dB cut-off level). Muscular evoked potentials (MEPs) were recorded bilaterally from subcutaneously placed needle electrodes over the musculus extensor carpi radialis (ECR) (10 and 20 cm above the os carpi accessorium) and the musculus tibialis cranialis (TC) (10 and 20 cm above the medial malleolus). These electrodes were connected to the differential inputs of the physiological amplifiers of the measuring system. A ground needle electrode was placed subcutaneously in the neck at the right side of the horse. Multipulse TES was performed with 3 biphasic pulses per train (ppt), pulse width (pw) of 0.1 ms/ phase and interpulse interval (ipi) of 1.3 ms. The stimulation voltage was increased using a stepwise protocol: 0, 2, 4, 6, 8, 10, 12, 14, 16, 18, 20, 22, 25, 30, 35, 40, 45, 50, 60, 70, 80, 90, 100, 110, 120, 130, 140, 150, 160, 170, 180, 190, 200 V. At each voltage, transcranial stimulation was performed twice. When transcranial motor thresholds (MT) were reached for both muscle groups, stimulation was continued to MT + 50 V or otherwise stopped at 200 V. At each occasion of stimulation, the motor responses were recorded for later retrieval of MEP amplitudes, MLTs and waveform morphology.

### MEP parameters

Considered parameters were: MLT, MEP amplitude and waveform morphology. The MLTs MLT_n,i,m_ were defined as ‘the time lag between the onsets of electrical stimulation and MEP response’ when these were unambiguously distinguishable from baseline noise level. Indices n, i and m refer to the n-th MEP, case number and muscle group in one of 4 extremities being ECR or TC. The amplitude A_n,i,m_. is the top-top amplitude of MEPs within the time window, between cessation of the stimulation artefact and onset of extracranially elicited late MEP responses. This time window precludes interference by extracranially elicited late MEP responses when analyzing transcranial MEPs [7]. We established a time delay of 0.7 ms between the trigger pulse and the onset of the stimulator trigger signal and actual start of the TES pulse train by an oscilloscope. The MLT values were subsequently corrected for the trigger delay. The waveforms are characterized by the number of phases.

The transcranial time window (TCW) is defined as ‘the time interval between the onsets of the transcranial and extracranial MEP components ’and computed at a TES voltage of MT + 20 V. For an in depth description of the determination of the transcranial time windows for the ECR and TC muscle MEPs we refer to the previously published scouting study [7]. MLTs and MEP amplitudes are analyzed within the TCW.

### Statistical analyses

Statistical analysis was performed with SPSS™ software, version 20.0.0, IBM™. A descriptive analysis was performed on the MLTs, amplitude and waveform morphology of the recorded MEPs. The normality of the relevant differences was graphically assessed using qq-plots. The conclusions of the graphical assessment were confirmed with a Shapiro-Wilk test. Overall, the assumption of normality could be accepted. For each case i and muscle group m, mean MLT_i,m_ and standard deviation SD_i,m_ were computed as 6 latency values of the latency data pairs from repeated stimulations at 10, 20 and 30 V above MT respectively. The MLT has its center point at MT + 20 V representing a common stimulation voltage that applies to the averages of 6 MLT values.

The reproducibility of the MLT measurements, expressing their intra- and inter-individual variability, was computed.

The mean MLT_m_, standard deviation SD_m_ and coefficients of variation, CV_m_, were computed from MLT_i,m_ and SD_i,m_ for all *N* = 12 cases. SD_intra,i,m_ is the intra-individual standard deviation and was used for computation of the mean intra-individual standard deviation mSD_intra,m_.

Left/right differences of MLTs and amplitudes were tested by a paired t-test with zero difference as a null hypothesis.

The voltage dependency of MLT_m_ was approximated by linear regression analysis. To exclude inter-individual variations of the MLTs, the 6 × 12 = 72 values were subtracted by MLT_i,m_ prior to further processing. Theoretically, the expectation value of zero is at MT + 20 V.

The dependency of MLT_m_ values on the height at withers was approximated by linear regression analysis.

A significance level of *p* ≤ 5% and confidence interval of 95% was applied throughout the study.

## Results

The measurements were successfully performed in all horses on all four limbs.

### Recorded normative values for MEP parameters

Table 1 provides an overview of normative data for MLTs, MEP amplitudes and waveform morphology recorded in ECR and TC muscles on the left and right side. MLTs showed no left versus right differences. Values of MLT were between 16.1–22.6 ms for the ECR and 31.9–41.1 ms for the TC muscles and were statistically equal within 95% confidence intervals. Intra-individual coefficients of variation, CV, for MLTs were low; their range and mean being 2.2–8,2% and 4.5% for the ECR and 1.4–6.3% and 3.5% for the TC. Inter-individual CVs for MLTs were higher, however below 10% on all occasions.

**Table 1:** Overview of the MLTs, MEP amplitude and number of phases per muscle group

Muscle group			ECR		TC	
			left	right	left	right
MLT (ms)	m		19.70	19.10	36.17	36.32
	SD | CV		1.48 | 0.075	0.83 | 0.042	2.12 | 0.059	2.40 | 0.066
	SD_intra_ | CV_intra_		0.83 | 0.042	0.89 | 0.047	1.23 | 0.051	1.21 | 0.033
	m-1.96 SD		16.8	16.1	31.9	31.5
	m + 1.96 SD		22.6	22.1	40.4	41.1
MEP amplitude (mV)	m		3.61	4.53	2.66	2.55
	SD | CV		2.55 | 0.71	3.10 | 0.68	2.22 | 0.83	1.85 | 0.73
	paired difference left-right	m	−0.93		0.11	
		SD	1.15		1.10	
		sig	0.017*		0.75	
Number of phases	biphasic		7	5	3	6
	triphasic		3	6	7	3
	four-phasic		2	1	1	1
	polyphasic (> 4)		–	–	1	1

MLTs are characterized by mean (m), standard deviation (SD), coefficient of variation (CV) mean intra-individual standard deviation (SD_intra_) being the average of SD_i_ over 12 cases, where SD_i_ is the standard deviation belonging to MLT_i,_ and mean ±1.96 SD delineating 95% probability ranges. MEP amplitudes are given as mean (m), SD and CV together with the mean (m), standard deviation (SD) and significance (sig) of paired MEP amplitude differences between the left and right muscle groups. CV is the coefficient of variation of mean MLTs. CV_intra_ is the coefficient of variation of mean paired MLT differences. ^*)^ significant for *p* ≤ 0.05

^*)^significant for *p* ≤ 0.05

Only in the thoracic limb muscle groups the MEP amplitudes showed a left-right difference (*p* < 0.05) in favor of the right side.

MEP waveforms were 2- or 3-phasic in about 80% of cases, regardless the muscle group. The remaining 20% were polyphasic, while a single MEP in the right TC muscle group was monophasic.

### Transcranial time windows

The 95% confidence intervals of the TCW were 15.8–19.0 ms (left) and 15.7–20.3 ms (right) for the ECR and 14.9–18.0 ms (left) and 15.1–18.4 ms (right) for the TC.

### Analyzed correlations

**Fig. 2:**
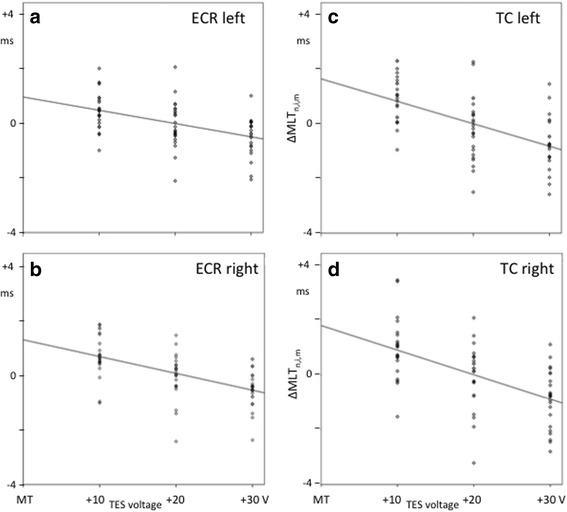
Scatter plots showing the correlation between motor MLT and TES intensity. Considered are changes of MLT due to increases in TES-voltage. Motor latency differences ∆MLT_n,i,m_ are plotted vertically and the TES-intensity is related to the motor threshold MT and plotted along the horizontal axis as V_TES_ – MT. ∆MLT_n,i,m_ is obtained after subtraction of the mean MLT, MLT_i,m_, from MLT_n,i,m_. n denotes case number, i is one of 6 data points per case and m refers to the muscle group. Each plot represents a muscle group: figures (**a**) and (**b**) refer to ECR: extensor carpi radialis muscle, respectively left and right. Figures: (**c**) and (**d**) refer to TC: tibial cranial muscle, respectively left and right. The parameters of the regression line with correlation and significance are specified in Table 2. All regression lines show decreasing courses with significant correlation

There was a clear voltage dependent decrease of MLTs, as depicted in Fig. 2 and Table 2.

**Table 2:** Overview of characteristic parameters of the regression lines of the MLT and TES-voltage

Muscle group		∆MLT = a + b (V_stim_ – MT)		Correlation	Significance
		a [ms]	b [ms/V]		
ECR	left	0.971	−0.049	0.480	.000^*^
	right	1.271	−0.061	0.559	.000^*^
TC	left	1.637	−0.082	0.566	.000^*^
	right	1.788	−0.089	0.563	.000^*^

*ECR* extensor carpi radialis, *TC* tibialis cranialis, *∆MLT* MLT difference as function of V_stim_–MT. Number of included values: 72 = 12 cases × 6 observations per case

*significant for *p* ≤ 0.05

All correlation factors were significant in the 4 muscle groups and most pronounced for the pelvic limb muscles. The slopes of the regression lines of the thoracic limb latency times were less steep with coefficients of − 0.049 (left) and − 0.061 ms/V (right), compared to those of the pelvic limb muscles, with coefficients of − 0.082 (left) and − 0.089 ms/V (right). No left-right differences were found. For a stimulation voltage increase of 20 V, the reduction in MLTs for the ECR was about − 1.5 ms and − 2.5 ms for the TC muscles. The reproducibility of a latency measurement is computed as the average of the standard errors of mean of the 12 cases. This yielded for the ECR: 0.34 ms (left), 0.36 ms (right) and bilateral mean: 0.35 ms, and for the TC: 0.50 ms, (left), 0.53 (right) and bilateral mean 0.52 ms.

**Fig. 3:**
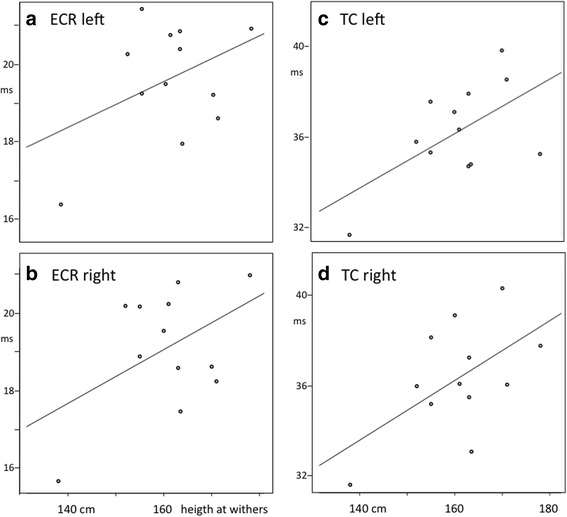
Scatter plots of MLT_i_ as a function of the height at withers. Scatter plots of MLT_i_ as a function of the height at withers where i refers to the case number. MLT_i_ is the mean MLT of 3 data pairs at stimulation voltages V_stim_ of 10, 20 and 30 V above motor threshold MT. One point represents the mean value of one case. All 12 cases are included. Each plot represents a muscle group. Figures (**a**) and (**b**) refer to ECR: extensor carpi radialis muscle, respectively left and right. Figures (**c**) and (**d**) refer to TC: tibial cranial muscle, respectively left and right. The parameters of the regression lines are specified in Table 3. All regression lines show increasing courses of which the left TC muscle group is significant for *p* ≤ 0.05

With respect to height at withers, only a significant correlation with MLTs for the left TC muscle was found and none for amplitudes (Fig. 3 and Table 3).

**Table 3:** Overview of the characteristic parameters of the regression lines of the MLT and height at withers

Muscle group		MLT = a + b _*_ withers		Correlation	Significance
		a [ms]	b [ms/cm]		
ECR	left	10.708	0.060	0.415	0.181
	right	8.531	0.070	0.465	0.128
TC	left	17.691	0.119	0.579	0.049^*^
	right	15.993	0.131	0.561	0.058

*ECR* extensor carpi radialis, *TC* tibialis cranialis, *MLT* is a function of the height at withers where a is the intercept and b the slope expressing increase of MLT per increase of height at withers. 12 cases are included

^*^Significant for *p* ≤ 0.05

The coefficients of the regression lines are listed in Table 3.

## Discussion

The goal of the current study was to provide normative data on TES induced MLTs, amplitudes and waveform morphology in the m. extensor carpi radialis and the *M. tibialis* cranialis bilaterally in a group of healthy horses. Furthermore, TES induced MEPs were checked for their intra-and inter individual reproducibility. The MLTs of the induced MEPs were checked for their stimulation voltage dependency. The influence of body side (left versus right) and height at withers of the horses was studied for both amplitude and MLTs. TES-muscle induced MEP studies have been used in different animal species like pigs, monkeys, cats, dogs and rabbits [5, 16, 22–25]. To our knowledge, this is the first study providing this data in healthy horses, when using the TES technique [7]. Mayhew et al. [1] published the first normative data on MLTs in ponies, and later Nollet et al. [3] in horses, using the TMS technique.

Our study shows that TES -a method currently used in intra-operative spinal function monitoring to promptly warn of impending damage to the nervous system in human patients subjected to brain or spinal surgery- is a promising technique to assess spinal motor function in horses.

The technique is easily applied. In our previous and current study, we showed that TES is painless, and usually well tolerated, in horses. The technique appears less sensitive to cortical function due to direct stimulation of the corticospinal tract [7]. Like in TMS, horses need to be sedated for reasons of safety.

### Comparison of MEP characteristics between TES and TMS

Mayhew et al. [1] reported mean MLTs of 19.0 ms for the ECR, and 30.2 ms for the TC muscles. Data in our study are quite similar. Therefore, based upon normative values published in literature on TMS, no significant difference between the two techniques can be claimed at this point.

Several factors hamper comparison between reported TMS normative data with the normative data on TES induced MEPs obtained in the current study. One is the dependence of MLTs on the height at withers. The data of Nollet et al. [3] is based on a height at withers of 137.8 ± 27.07 cm, which is marked lower than in our study. Nollet et al. [3] depict at 160.8 cm (mean height in our study) MLTs of 21.2 ms for the thoracic and 34.1 ms for the pelvic limb muscles. This was in our study 1.8 ms lower for the ECR and 2.1 ms higher for the TC muscles. The mentioned insecurities limit comparisons within 2–3 ms accuracy. This marge is too large to detect the small differences up to 2 ms in MLTs from TES and TMS. These small differences of MLTs would only be detectable by pairwise comparisons of TES and TMS used together in individual horses [9, 16, 26, 27].

### Reproducibility of the technique.

Coil positioning in TMS has shown to significantly change MLT, which is not the case for TES as the electrodes stay in the same position. According to Nollet et al. [2] the effect of coil repositioning errors on MLTs is most critical in lateral directions. According to Table 1, both intra- and inter-individual coefficients of variation of MLTs were quite low: 0.059–0.081 and 0.033–0.051 respectively. Intra-individual coefficients of variation especially showed low values, underlining a good reproducibility of repeated measurements within the same horse, when using the TES technique.

Furthermore, TES predominantly bypasses the brain cortex by immediate activation of corticospinal tracts located in deeper regions. This minimizes motor cortical influence and possible influence of sedatives and anesthesia in the neural transmission across the UMN and thus may enhance reproducibility [5].

### Comparison of MEP characteristics between sides.

Left/right differences were not found for any of the observed MEP characteristics, with the exception of the reported paired left/right difference for the recorded thoracic MEP amplitude (Table 1) with left being 3.61 ± 2.55 mV, and right: 3.10 ± 3.10 mV. Future studies including more horses may help to elucidate this issue.

### Transcranial time window.

It is important to delineate a transcranial time window in which transcranial MEPs can be isolated to avoid interference with late MEPs that most likely result from reflexes that are elicited from extracranial current conduction [7]. As in previous studies [1, 2, 28, 29] late MEPs were also seen in all horses included in the current study. This occurrence of late MEPs is unique for horses. In human, similar effects are seen in a hyperactive spinal cord often resulting from cranially located lesions in the spinal cord or the brain. As previously proposed, these late MEPs in horses result most probably from reflex circuits, which are elicited by stimulation of extracranially located sensory axons [7]. The earlier manifestation of the transcranial MEPs of interest leaves a time window enabling a selective analysis of MEPs without contamination of MEPs from another origin. The window is about 16–20 ms in ECR and 15–18.5 ms in TC muscles (95% confidence interval). It is important to realize that the duration of transcranial MEPs may exceed this defined time window so that interpretation of MEP characteristics in the last part of polyphasic patterns of the MEP wave outside the window may become inaccurate. These interfering effects outside the transcranial time window on phases pertain to TES and also TMS techniques. This will likely complicate its clinical interpretation in pathological conditions of the spinal cord.

### Voltage dependency of MLTs

TES voltage dependent decreases of MLTs are visible in d-waves [30, 31]. Similar MLT decreases are also reported in muscle MEPs of dogs [32]. Table 2 shows comparable data in horses. Figure 2 shows the gradual decrease of MLTs in function of the applied TES voltage, for each extremity. These MLT reductions were observed in each horse individually without exceptions. The average decrease of MLTs over MT + 10 V to MT + 30 V in the thoracic limbs is about − 1 ms and − 1.7 ms in the pelvic limbs. A further decrease to nearly − 3 ms is achieved at MT + 50 V. The MLT reduction can partly be explained by deeper activation of motor tracts at higher stimulation intensities. This causes an earlier start of the epsp summation process at LMNs. The much larger mean muscular MLT reduction of − 7,45 ms at increasing TMS intensities of Nollet et al. [19], resembles the latency jumps in the transition region from extracranial to intracranial elicited MEPs in our study. In that study, no transcranial time window was considered. This entails that the reported MLT averages comprise a mix of transcranial and extracranial MEP components in all muscle groups. Therefore, this study is the first to report on stimulation voltage dependency of MLTs by taking the proposed transcranial time window into account.

One can anticipate on the intensity bias of MLTs of − 1 to − 2 ms by choosing the default intensity at fixed voltage above MT, e.g. MT + 20 V, or at fixed percentage above MT.

### Influence of height at withers on TES induced MLTs

A trend of positive correlation was found between height at withers and MLTs in all 4 muscle groups (Table 3). However, only the correlation of the left TC MEPs with height at withers was significant. Nollet et al. reported that height at withers correlated well with the length of motor tracts in the spinal cord and peripheral nerves [3]. The correlation is ascribed to the linear relation between conduction times and axon length, which in turn is related to geometric dimensions of the horse when assuming equal action potential conduction velocities. Our observations are in reasonable agreement with those findings in TMS studies [3]. However, the number of horses in our study was probably too small to offer enough significant statistical power to demonstrate significant correlations in all 4 muscle groups, which is a limitation of our study.

### Temperature effects

Axonal conduction velocities and related MLTs depend on temperature. The part of the motor conduction route that is involved in the TES procedure is mainly embedded in tissues well within central body temperature range. MLT changes under hypothermia in TES studies in pigs and rabbits are in a range of − 1 to − 5% at an increase of body temperature of 1 °C [22, 23]. In the normal range of body temperature in horses (37.4–38.0 °C), the expected inter-individual variation in MLT are for the ECR − 0.12 ms to − 0.5 ms and for the TC -0.22 ms to − 1 ms. This is well within the inaccuracy of transcranial MLTs.

## Conclusions

TES is a novel and sensitive technique to assess motor function in horses. It is easily applied and highly reproducible. The current study provides normative data in healthy horses on TES induced MEPs in the extensor carpi radialis and tibialis cranialis muscles bilaterally. It is important to notice that extracranial elicited late MEPs appear to be a persistent side effect in horses undergoing electrical or magnetic transcranial stimulation, thus restricting reliable assessment of MEP characteristics to a transcranial time window of about 15–19 ms. For all MEP characterizing parameters no significant left to right differences were demonstrated. A significant effect of stimulation voltage on MLT’s was found. No significant effect of height at withers could be found based upon the results of the current study. A study in which both TMS and TES are applied on the same group of horses is needed.

## Abbreviations

A_n,i,m_: Amplitude for the n-th MEP, case i and muscle group m
CV: Coefficients of variation
ECR: Musculus extensor carpi radialis
epsp: Excitatory postsynaptic potential
i: Case
LMN: Lower motor neuron
m: Muscle
MEP: Motor evoked potential
MLT: Motor latency time
MLT_m_: Mean motor latency time
MLT_n,i,m_: Motor latency times n denotes case number, i is one of 6 data points per case and m refers to the muscle group
mSD_intra, i, m_: Mean intra-individual Standard Deviation
MT: Motor thresholds
n: n-th MEP
SD_intra, i, m_: Intra-individual Standard Deviation
SD_m_: Mean Standard deviation
TC: Musculus tibialis cranialis
TCW: Transcranial time window
TES: Transcranial electrical stimulation
TMS: Transcranial magnetic stimulation
UMN: Upper motor neuron
